# Adults with obesity and hyperglycemia have higher arachidonic and docosahexaenoic acid in subcutaneous adipose tissue

**DOI:** 10.1016/j.jlr.2026.101106

**Published:** 2026-07-17

**Authors:** Sabrina Ziai, Mihir Parikh, Narjes Mosavari, Ruxandra D. Rotarescu, Chuck T. Chen, Richard P. Bazinet, André Tchernof, Anthony J. Hanley, Adam H. Metherel, Jacqueline L. Beaudry

**Affiliations:** 1 Department of Nutritional Sciences, Temerty Faculty of Medicine, University of Toronto, Toronto, Ontario, Canada; 2 School of Nutrition, Université Laval, Quebec City, Quebec, Canada

**Keywords:** Lipids, Omega-3 fatty acids, Triacylglycerol, Phospholipids, Fatty Acid Metabolism, Eicosapentaenoic acid

## Abstract

In obesity, fatty acid (FA) accumulation may impair AT function, promoting insulin resistance and development of type 2 diabetes mellitus (T2DM). However, the contribution of visceral AT (VAT) and subcutaneous AT (SAT) specific FAs to metabolic dysregulation in obesity and T2DM remains unclear, highlighting the importance of examining the FA composition between AT depots. This study examined differences in FA profile and lipid fractions across AT depots in individuals with obesity and hyperglycemia. The primary focus was on polyunsaturated fatty acids (PUFAs) given their relevance to cardiometabolic health. Thirty participants matching VAT and SAT samples from bariatric surgery donors (BMI ≥ 30 kg/m^2^) were examined and were further stratified into groups of fasted normoglycemia or hyperglycemia. For the total FA profile, the concentrations (μmol/g) of arachidonic acid (ARA), eicosapentaenoic acid (EPA), and docosahexaenoic acid (DHA) were higher in the SAT compared to VAT depot; ARA and DHA concentrations were higher in the hyperglycemia compared to normoglycemia group. Further analysis in six different lipid fractions including triacylglycerol, diacylglycerol, monoacylglycerol, free fatty acids, phospholipid and cholesteryl esters exhibited similarly higher ARA, EPA, and DHA in the SAT compared to VAT depot. However, differences between normoglycemia and hyperglycemia groups were fraction- and FA-specific, rather than uniform across all lipid classes. These results demonstrate that VAT and SAT depots differentially store FAs as a function of glycemic status in obesity, highlighting distinct differences in PUFA metabolism and availability that may have important implications for AT function and metabolic health.

Obesity is a chronic disease, caused by abnormal or excess body fat accumulation (adiposity), which impairs lipid and glucose homeostasis. It is a key risk factor for hyperglycemia, type 2 diabetes mellitus (T2DM), cardiovascular disease, and dyslipidemia (https://www.who.int/news-room/fact-sheets/detail/obesity-and-overweight) (1, 2). Metabolically healthy adipose tissue (AT) properly stores excess lipids and prevents ectopic fat deposition. However, impaired or metabolically unhealthy AT function is associated with lipid spillover, inflammation, and insulin resistance and may contribute to the pathological transition from normoglycemia to hyperglycemia in obesity. Notably, this dysfunction may be depot-specific, as AT is heterogeneous. Visceral AT (VAT) and subcutaneous AT (SAT) depots differ in anatomical, cellular, molecular, and physiological characteristics, which contribute to varying adipokine secretion, insulin sensitivity and lipid handling (3, 4, 5). In comparison to SAT, VAT is more cellular, vascularized and contains a higher number of immune cells (6). SAT adipocytes have a higher capacity to expand and usually remain more insulin sensitive while taking up fatty acids (FAs), whereas VAT is more insulin resistant and exhibits higher lipolytic responsiveness (6). Collectively, these depot-specific differences suggest that excess VAT accumulation or impaired SAT storage capacity may underlie an increased risk of adverse metabolic outcomes (6).

AT dysfunction can influence the systemic FA profile by altering FA storage and release, thereby impacting both AT composition and circulating levels. Excess or dysregulated FAs can induce oxidative and endoplasmic reticulum stress, mitochondrial dysfunction and impaired insulin signaling (7, 8). These processes reduce glucose uptake in insulin-sensitive tissues, contributing to insulin resistance and resulting in hyperglycemia. Moreover, elevated circulating FAs may contribute to pancreatic beta-cell dysfunction and impair insulin secretion (9). Alterations in AT FAs may influence membrane fluidity, receptor function and intracellular signaling cascades that are involved in lipid metabolism and glucose uptake (7). Therefore, FA composition within AT may influence glycemic control by either adipocyte insulin responsiveness or interaction with the pancreas (10). Notably, omega-6 (n-6) and omega-3 (n-3) polyunsaturated fatty acids (PUFAs) are generally associated with pro- and anti-inflammatory effects, respectively. Major long-chain PUFAs include arachidonic acid (ARA; n-6) and the n-3 PUFAs eicosapentaenoic acid (EPA) and docosahexaenoic acid (DHA) (11). Importantly, n-3 PUFAs have been inversely associated with AT inflammation (12, 13), but the extent to which PUFAs in SAT and VAT depots are altered in obesity and hyperglycemia remains unclear.

Previous studies have primarily relied on circulating PUFA measurements (12, 14) rather than direct assessment of SAT and VAT composition, with the majority focusing on FA composition following dietary or supplemental interventions (15). Studies have also compared lean healthy controls to individuals with obesity (16), with or without T2DM, which may over-attribute FA differences to adiposity rather than underlying metabolic changes. Moreover, the link between FAs in ATs and whole-body glucose control needs further investigation. Therefore, the present study aimed to characterize FA composition in participant-matching plasma, VAT and SAT samples obtained from individuals with obesity and differing fasted glycemic status, while maintaining normolipidemia. By examining depot-specific FA profiles in human AT, we aim to identify compositional patterns of AT FAs and how they may associate with impaired glucose regulation, thus providing insights into AT lipid handling and its role in metabolic dysfunction. Our work is novel as it integrates plasma FA profile, AT total FAs and fraction-specific lipid pools, and inflammatory markers measured in both systemic circulation and AT depots using samples from the same individuals. No studies have differentiated lipid fraction-specific FA levels in human VAT and SAT depots, which sheds light on functional compartmentalization of lipids to better understand adipose lipid storage and metabolism. This work provides a unique comprehensive characterization of lipid distribution and inflammatory status in humans with obesity with differing glycemic states. Importantly, the inclusion of both plasma and AT measurements allows the evaluation of whether depot- and glycemia-specific FA differences are systemically reflected or tissue-restricted, alongside preliminary assessment of whether these changes align with inflammatory alterations.

## Materials and methods

### Ethics statement

The study protocol #00046404 was approved by the Human Participant Ethics Protocol at the University of Toronto and complies with the Declaration of Helsinki.

### Study population characteristics

Thirty participant-matching plasma, VAT and SAT biopsies were obtained from individuals with a BMI of 30–45 kg/m^2^, who were bariatric surgery donors with normolipidemic status. Samples were collected from the Biobank at *Institut universitaire de cardiologie et de pneumologie de Québec, Université Laval*, Quebec, according to institutionally approved management modalities. All participants provided written, informed consent. The anthropometric and bioclinical measurements presented in Table 1 were obtained at the time of surgery and based on the fasting blood glucose and hemoglobin A1c (HbA1c); the patient samples were categorized into normoglycemia and hyperglycemia groups. Homeostatic Model Assessment for Insulin Resistance (HOMA-IR) measurements were taken by glucose (mmol/L) multiplied by insulin (μIU/ml) and divided by 22.5 (17). Plasma glucose measurements were completed in duplicate x 10 μl of sample using glucose analyzer (GM9-Analox). Plasma insulin measurements were run using a 4-fold dilution V-Plex® Human Insulin Assay (Cat#. K151S5-Series, LOT#. K0082754, Meso Scale Discovery®, Rockville, MD, USA). MESO QuickPlex SQ 120MM was used to read the plate, and data were analyzed on the Discovery WorkbenchTM 2013 LSR_4_0_13 (Meso Scale Discovery®).

**Table 1:** Bariatric surgery donor characteristics with normoglycemia and hyperglycemia

Parameters	Normoglycemia	Hyperglycemia	*t* test (*P*-value)
Anthropometric measurements			
Number of subjects	15 (3 males, 12 females)	15 (3 males, 12 females)	
Age (years)	47 ± 1.95	47 ± 2.11	0.9267
Weight (kg)	127.85 ± 8.35	126.54 ± 5.51	0.8970
Height (cm)	165.76 ± 3.39	164.07 ± 2.06	0.6730
BMI (kg/m^2^)	45.99 ± 2.05	46.98 ± 1.81	0.7208
Waist size (cm)	126.13 ± 10.48	132.83 ± 15.06	0.8329
Hip size (cm)	134.60 ± 10.58	141.27 ± 3.44	0.6866
Waist hip ratio	0.87 ± 0.06	0.94 ± 0.02	0.8522
Bioclinical measurements			
Fasting blood glucose (mmol/L)^a^	5.22 ± 0.17	7.49 ± 0.56	<0.0001^d^
Plasma glucose (mmol/L) (n = 14)	5.00 ± 0.15	6.53 ± 0.49	0.0062^c^
Plasma insulin (μIU/ml) (n = 14)	29.52 ± 6.79	47.02 ± 9.18	0.1509
HbA1c (%)	4.97 ± 0.0004	6.17 ± 0.006 (n = 13)	<0.0001^d^
HOMA-IR (n = 14)	6.37 ± 1.33	12.68 ± 2.24	0.0229^b^
Total cholesterol (mmol/L)	4.57 ± 0.17	5.00 ± 0.21	0.1152
HDL (mmol/L)	1.46 ± 0.09	1.26 ± 0.09	0.1002
LDL (mmol/L)	2.45 ± 0.14	2.99 ± 0.18	0.0245^b^
Triglycerides (mmol/L)	1.45 ± 0.17	1.65 ± 0.12	0.3328

Data represented as mean ± SEM. A student *t* test was performed to determine significance between groups.

a The fasting blood glucose was measured several weeks prior to the bariatric surgery.

b *P* < 0.05.

c *P* < 0.01.

d *P* < 0.0001.

### Lipid extraction from plasma and adipose tissue

Plasma samples were thawed and 150 μl was used for the lipid extraction. VAT and SAT samples were weighed (∼20 mg) and pulverized on dry ice using a tissue pulverizer (Spectrum™ Bessman), and lipids were extracted in glass tubes 16 × 100 MM (cat# 1495935AA, Fisherbrand) using the Folch method (18), as previously modified (19). The samples were extracted in 3 ml of 2:1 chloroform (Supelco cat# CX1055-9, Millipore-Sigma, ON, Canada): methanol (cat# 179337-4L, Millipore-Sigma, ON, Canada) (v:v) containing 50 μg of docosatrienoic acid (22:3n-3) ethyl ester as an internal standard (Nu Chek Prep, Inc., Elysian, MN) for total FA determinations. Phase separation between the organic and aqueous layers was facilitated by adding 1 ml of 0.88% KCl (cat# P9541-500G, Millipore-Sigma, ON, Canada), followed by centrifugation at 500 x g for 5 min at 4°C. The bottom chloroform layer containing total lipid extract (TLE) was isolated and an aliquot of 1200 μl of plasma and 200 μl of AT was dried by nitrogen gas using a nitrogen generator (Peak Scientific Genius XE Nitrogen, Inchinnan, Scotland) and evaporator (Reacti-therm III #TS-18824, Thermo Scientific). TLE were then transesterified with 1 ml of 14% boron trifluoride in methanol (cat# B1252-1L, Millipore-Sigma) and 300 μl of hexane (cat# 178918-4L, Millipore-Sigma, ON, Canada) in an oven for 1 h at 100°C (Mod: PR305220M Thermo Scientific, OH, USA) to fatty acid methyl esters (FAMEs) (20). Samples were placed on the benchtop to cool to room temperature (22°C) and 1 ml of hexane and 1 ml of double-distilled water (Milli-Q, Fisher Scientific, ON, Canada) were added, vortexed and centrifuged for 5 min at 500 x g at 4°C. The top hexane layer containing FAMEs was isolated and dried using nitrogen gas and then reconstituted in 100 μl of heptane (cat# O3008-4, Fisher chemical). The samples were then analyzed by gas chromatography-flame ionization detection (GC-FID, SCION 8300) for FA levels. A pilot quality control experiment was performed using butylated hydroxytoluene (BHT) to assess potential FA oxidation during the lipid extraction. No differences were observed between samples processed with or without BHT, indicating lipid extraction procedures did not result in detectable FA oxidation.

### Thin-layer chromatography

The samples were prepared as described in the lipid extraction section, using the same equipment and chemical brands. However, the internal standards for thin-layer chromatography (TLC) were heptadecanoic acid (17:0)-containing phosphatidylcholine (PL) (28 μg) (Avanti Polar Lipids Inc), TAG (84 μg), DAG (22 μg), MAG (26 μg), FFA (20 μg) and CE (47 μg) (Nu-Chek Prep, Inc) per sample. This equates to approximately 20 μg of 17:0 in all internal standards with the exception of TAGs, which included ∼80 μg of 17:0. An aliquot of the TLE samples was dried down using nitrogen gas and reconstituted in 100 μl chloroform. The 20 × 20 cm, 250 mm G-TLC plate (Uniplate™ Analtech Silica gel G TLC Plate Miles Scientific, Fisher Scientific, ON, Canada) was scored, 1 cm for reference standard lanes and 1.5 cm for samples and placed in the TLC glass developing tank with 100 ml of chloroform:methanol mix (1:1), and washed overnight prior to use. The scored washed plate was then placed in the oven 100°C for 1 h to be activated. For preparing the TLC tank, 60 ml heptane (cat# O3008-4 Fisher chemical, Fisher Scientific), 40 ml diethyl ether (cat# 4700-1-10 Caledon Laboratory Chemicals), 2 ml glacial acetic acid (cat# ACE333.500 BioShop® Inc.) were added. For the TLC standards reference and samples, 50 μl were loaded to the plate. The TLC plate was placed into the prepared tank, and the solvents ran to approximately 1 inch from the top of the plate. The plate was then removed from the tank and placed on plate holder to dry (∼1 min). It was then sprayed with 0.1% 8-Anilino-1-naphthalenesulfonic acid (ANSA) (0.1 g per 100 ml water) in the fume hood, and the bands were visualized under UV light (Model CC 10 Ultra-violet products Inc) in the dark. The TLC lipid bands were scored, scraped and collected into labelled test tubes, and transesterified to FAMEs as described earlier. The FAMEs were then reconstituted in 100 μl of heptane for all fractions besides TAGs which were in 200 μl and stored in GC vials until GC-FID analysis.

### Gas chromatography

FAMEs were analyzed using GC-FID, which was equipped with a DB-FFAP 15 m × 0.10 mm i.d. × 0.10 μm film thickness, nitroterephthalic acid modified, polyethylene glycol, capillary column (J&W Scientific from Agilant Technologies) (21). An autosampler, (SCION 8400 PRO, SCION instruments, Goes, NL) injected 1 μl of FAMEs into the GC-FID with the injector heated at 250°C with a split ratio of 200:1 for TAGs and 60:1 for all other lipid fractions. The oven temperature started at 150°C with a 0.25 min hold, followed by a 35°C/min ramp to 200°C, a 4°C/min ramp to 225°C with a 2.07 min hold, and then an 80°C/min ramp up to 245°C with a 13 min final hold at the end. The detector was maintained at 300°C, with air and nitrogen gases flowing at 300 ml and 30 ml/min, respectively, and a sampling frequency of 100 Hz. Retention times were used to identify peaks by comparison to an external mixed FAME standard (GLC-462, Nu Chek Prep, Inc., Elysian, MN). The resulting chromatograms were analyzed using Compass CDS software (Version 3.0.0.68), and concentrations (μmol/g) of FAMEs were quantified by dividing the area under the curve of the FAME peak by the internal standard peak and multiped by the known amount of internal standard. Units for concentrations were corrected for AT weight and individual FA molar mass.

### Inflammatory markers

Plasma C-reactive protein (CRP), a marker of systemic inflammation (22), was assessed using U-Plex® Human CRP Assay (MesoScale Discovery, Cat# K151L9K-1, LOT# 558011). Monocyte chemoattractant protein-1 (MCP-1) involved in immune cell and macrophage infiltration (23) was assessed using U-Plex® Custom Metabolic Group 1 (hu) Assays (MesoScale Discovery, Cat# K151ACM-1, LOT# 534058). Cluster of differentiation 80 (CD80), an immune cell activator (24) and interleukin-8 (IL-8), a pro-inflammatory cytokine (25), were assessed using U-Plex® Custom Immuno-Oncology Grp 1 (hu) Assays (MesoScale Discovery, Cat# K151AEM-1, LOT# 533472). Adiponectin, an insulin sensitivity marker (26) was assessed using the U-Plex® Human Adiponectin Assay (MesoScale Discovery, Cat# K151R9K-1, LOT# 554430). In AT, adiponectin was assessed using the U-Plex® Human Adiponectin Assay (MesoScale Discovery, Cat# K151R9K-1, LOT# 533473), MCP-1 levels were assessed using the U-Plex® Custom Metabolic Group 1 (hu) Assays (MesoScale Discovery, Cat# K151ACM-1, LOT# 533471) and IL-8 levels using U-Plex® Custom Immuno-Oncology Grp 1 (hu) Assays (MesoScale Discovery, Cat# K151AEM-1, LOT# 534056). The plates were read and data analyzed on the instrument and software mentioned previously in the methods. Plasma samples were thawed once before being diluted 16,000-fold for CPR, 2-fold for MCP-1, CD80 and IL-8 and 400,000-fold for adiponectin using the metabolic assay solution prepared with assay diluent. VAT and SAT homogenates were prepared using 50 mg of tissue in 1 ml of the MSD lysis buffer (150 mM NaCl, 20 mM Tris pH 7.5, 1 mM EGTA, 1 mM EDTA, 1% Triton X-100) using TissueLyser II, (Qiagen, USA) and a standard Bradford assay (Bio-Rad Cat# 5000001, Mississauga, Ontario, Canada) was performed to determine protein concentrations. The AT homogenates used for adiponectin were diluted to a 200,000-fold, and for MCP-1 and IL-8, they were diluted to a 2-fold factor. For both plasma and AT analytes, 50 μl of samples were loaded on assay plates, and the experiments were performed based on the manufacturer's protocol.

### Statistical analyses

Student’s *t* tests were performed on the patient characteristics described in Table 1, plasma FA data in Supplemental Table S1 and plasma inflammatory markers in Supplemental Table S16. For FA profiles of total lipids, lipid fractions and inflammatory markers in AT samples, two-way ANOVAs were performed, and if an interaction was present, Tukey’s multiple comparisons test was conducted in GraphPad Prism 10 (GraphPad Software). Normality of model residuals was assessed using Shapiro-Wilk, and when necessary, data were log-transformed to approach normality prior to statistical analysis. The statistical significance was set at *P* ≤ 0.05 and the outlier tests were performed using Grubb’s test using the outlier calculator with the significance threshold of α = 0.05. Approximately one to two observations were identified as outliers per fatty acid and in the inflammatory markers. Outliers identified in either plasma glucose or insulin were removed and not included in the calculated HOMA-IR. These are explicitly mentioned in Table 1. None of the outlier exclusions affect the main findings.

## Results

### Fatty acid concentrations in plasma and human adipose tissues

#### Total lipid pool

**Fig. 1:**
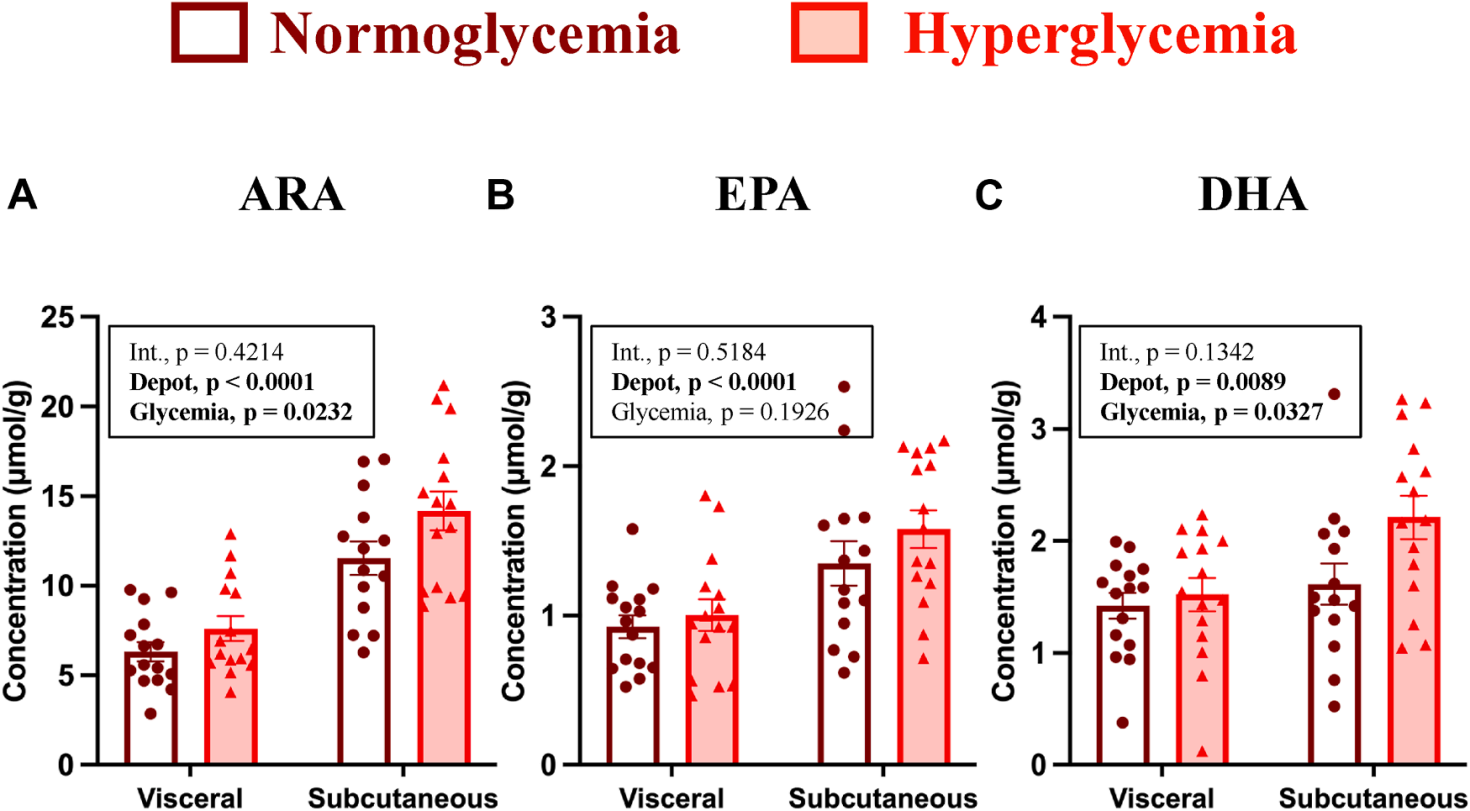
Fatty acid concentrations (μmol/g) in the VAT and SAT depots between normoglycemia and hyperglycemia groups. A: Arachidonic acid (ARA); (B) eicosapentaenoic acid (EPA), and; (C) docosahexaenoic acid (DHA). Data presented as mean ± SEM were analyzed using two-way ANOVA followed by Tukey’s post hoc test if there was an interaction effect, ∗*P* < 0.05, ∗∗*P* < 0.01, ∗∗∗*P* < 0.001 ∗∗∗∗*P* < 0.0001. N = 14–15 per group.

To characterize overall FA profile, total lipid FA concentrations (μmol/g) were assessed in participant-matching plasma, VAT and SAT depots across normoglycemia and hyperglycemia groups. While the FA profile quantified included saturated FA (SFA) and monounsaturated FA (MUFAs) (data presented in Supplemental Tables S1–S15), our analysis focused on PUFAs given their well-established roles in inflammation and cardiometabolic disease, which were the primary outcomes of interest. Although other FAs contribute to whole-body function, PUFAs are particularly relevant because they serve as precursors to lipid mediators. For plasma, no differences were observed in any FA assessed based on level of glycemia (Supplemental Table S1). Additionally, there were no interaction effects within the total AT lipid pool (depot × glycemia) for ARA (*P* = 0.4214), EPA (*P* = 0.5184) and DHA (*P* = 0.1342) concentrations. However, there were main effects of depot where ARA (*P* < 0.0001, Fig. 1A), EPA (*P* < 0.0001, Fig. 1B) and DHA (*P* = 0.0089, Fig. 1C) were all higher in SAT compared with VAT. In addition to these PUFAs, several others such as *γ*-linolenic acid, dihomo-*γ*-linolenic acid and n-3 docosapentaenoic acid were also present in higher concentrations in the SAT depot (Supplemental Table S2). Main effects of glycemia indicated that ARA (*P* = 0.0232) and DHA (*P* = 0.0327) were both higher in hyperglycemia compared with normoglycemia by approximately 19%, and 27%, respectively. There was no main effect of glycemia (*P* = 0.1926) on EPA. Briefly, despite no interaction, main effects of depot were observed, with total concentrations of 20:0 (*P* < 0.0001), 22:0 (*P* = 0.0183), 20:1n-9 (*P* = 0.0105), 24:1n-9 (*P* = 0.0008) and 18:4n-3 (*P* < 0.0001) being lower in SAT compared to VAT depot. Main effects of glycemia were also observed, with 23:0 (*P* = 0.0401), 14:1 (*P* = 0.0386), and 22:1n-9 (*P* = 0.0030) being lower in those with hyperglycemia compared to the normoglycemia group by approximately 40%, 26%, and 24%, respectively.

To determine which specific lipid pools contributed to the consistently observed differences in total ARA, EPA and DHA in the total lipids, FA concentrations were assessed across six lipid fractions including TAG, DAG, MAG, PL, FFA, and CE.

### Arachidonic acid in adipose tissue lipid fractions

**Fig. 2:**
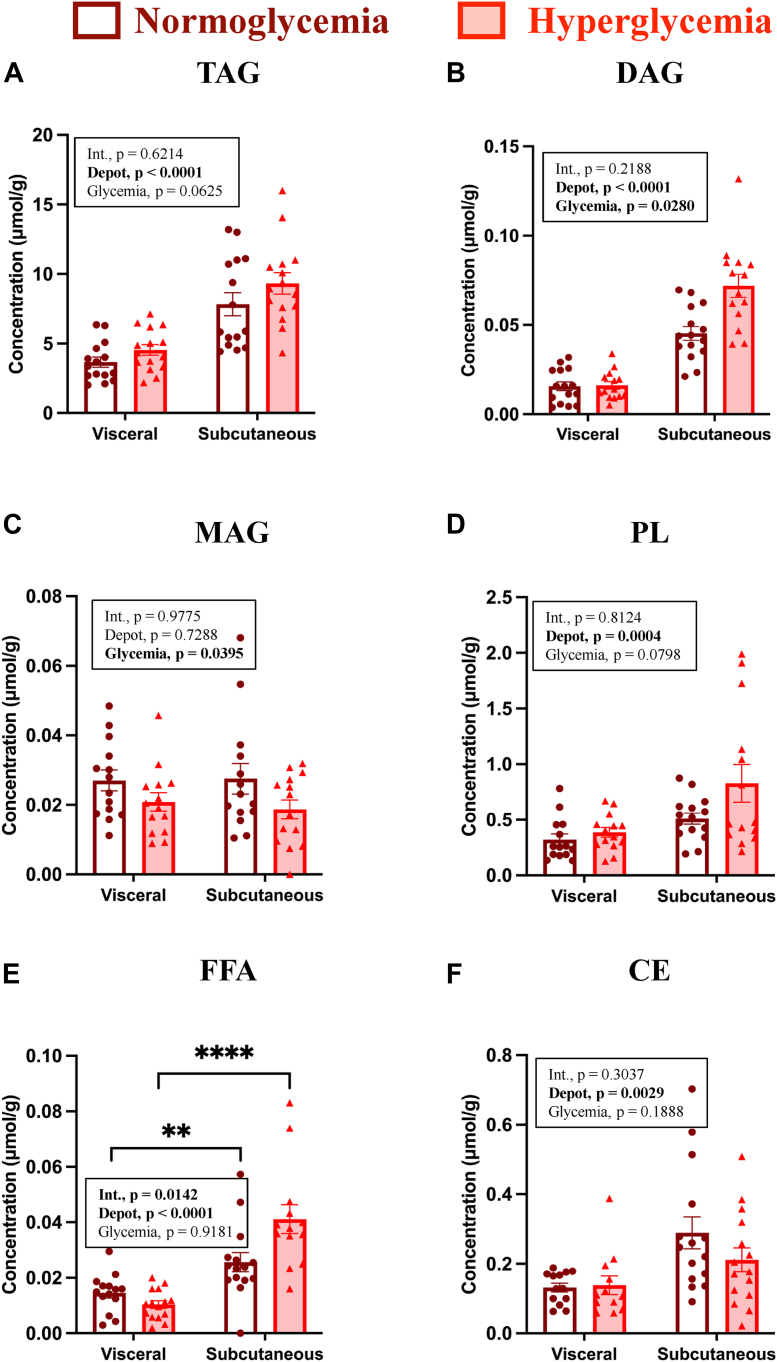
Arachidonic acid (ARA) concentrations (μmol/g) in the VAT and SAT depots in between normoglycemia and hyperglycemia groups in different lipid fractions. A: Triacylglycerol (TAG), (B) diacylglycerol (DAG), (C) monoacylglycerol (MAG), (D) phospholipids (PL), (E) free fatty acids (FFA) and (F) cholesteryl esters (CE). Data presented as mean ± SEM were analyzed using two-way ANOVA followed by Tukey’s post hoc test if there was an interaction effect, ∗*P* < 0.05, ∗∗*P* < 0.01, ∗∗∗∗*P* < 0.0001. N = 13–15 per group.

Lipid fraction data are presented based on the main effects of depot and glycemic status; interactions, where present, are stated explicitly. ARA concentrations were higher in TAG (*P* < 0.0001), DAG (*P* < 0.0001), PL (*P* = 0.0004), and CE (*P* = 0.0029) fractions in the SAT compared to the VAT depot (Figs. 2A, B, D, F). The hyperglycemia group had ∼ 37% higher ARA in DAG (*P* = 0.0280, Fig. 2B) compared to normoglycemia group. ARA in MAG fraction was ∼ 33% lower in hyperglycemia group compared to normoglycemia group (*P* = 0.0395, Fig. 2C). An interaction effect was present in the FFA (*P* = 0.0142, depot × glycemia) fraction, indicating that the effect of depot differed by glycemic status and vice versa (Fig. 2E).

### Eicosapentaenoic acid in adipose tissue lipid fractions

**Fig. 3:**
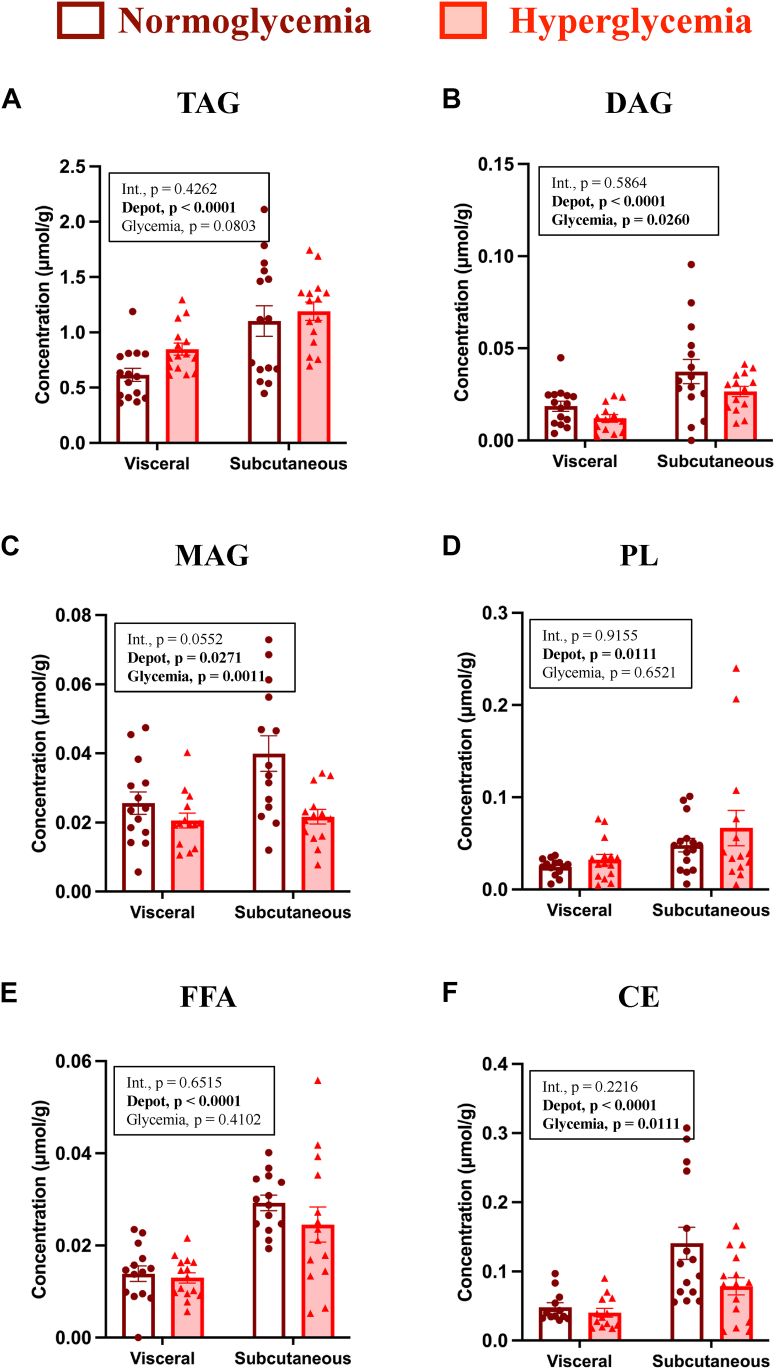
Eicosapentaenoic acid (EPA) concentrations (μmol/g) in the VAT and SAT depots in between normoglycemia and hyperglycemia groups in different lipid fractions. A: Triacylglycerol (TAG), (B) diacylglycerol (DAG), (C) monoacylglycerol (MAG), (D) phospholipids (PL), (E) free fatty acids (FFA), and (F) cholesteryl esters (CE). Data presented as mean ± SEM were analyzed using two-way ANOVA followed by Tukey’s post hoc test if there was an interaction effect, ∗*P* < 0.05, ∗∗*P* < 0.01, ∗∗∗*P* < 0.001. N = 13–15 per group.

EPA concentrations were higher in all six fractions including TAG (*P* < 0.0001), DAG (*P* < 0.0001), MAG (*P* = 0.0271), PL (*P* = 0.0111), FFA (*P* < 0.0001) and CE (*P* < 0.0001) in SAT compared to VAT depot (Fig. 3A–F). In terms of glycemic status, EPA was lower in the DAG (*P* = 0.0260), MAG (*P* = 0.0011) and CE (*P* = 0.0111) in the hyperglycemia group compared to normoglycemia group by approximately 28%, 45%, and 45%, respectively (Figs. 3B, C, F).

### Docosahexaenoic acid in adipose tissue lipid fractions

**Fig. 4:**
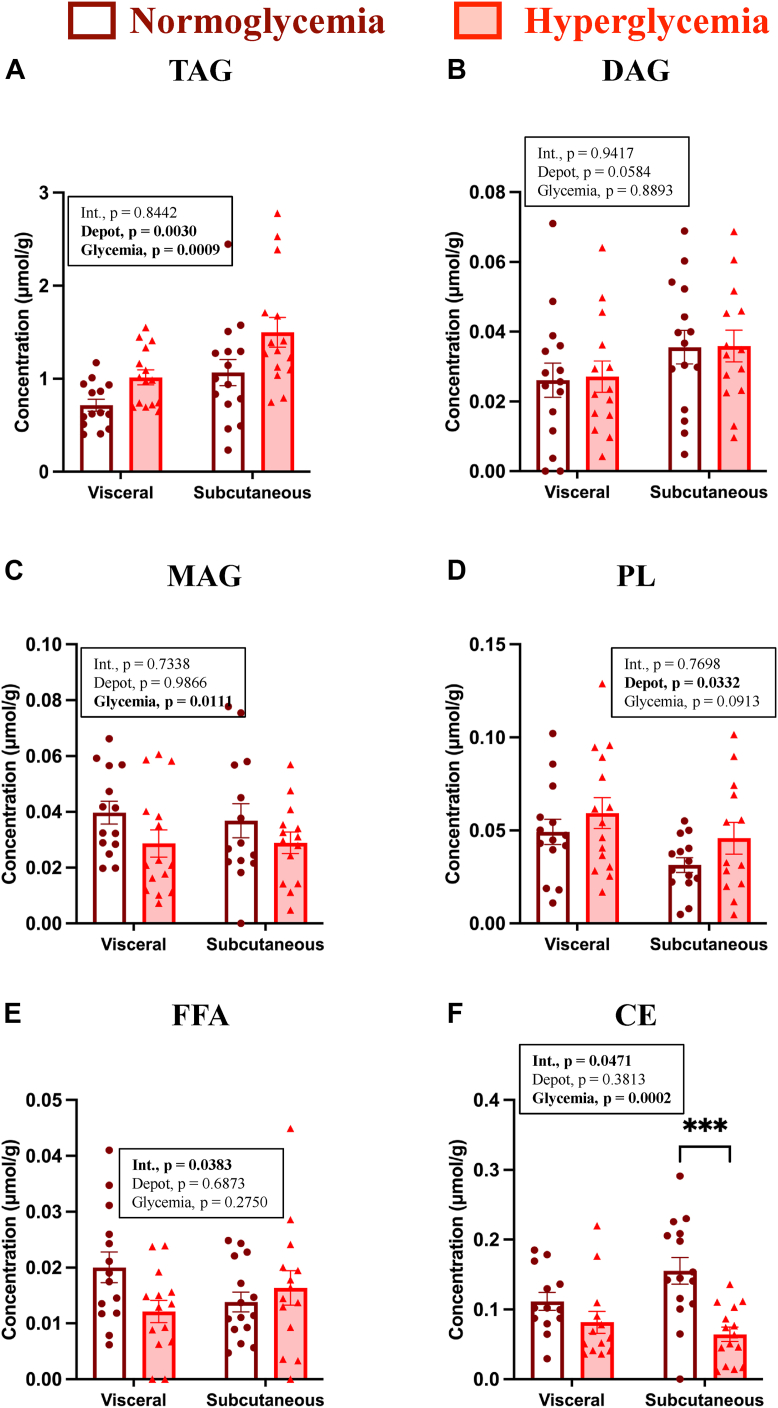
Docosahexaenoic acid (DHA) concentrations (μmol/g) in the VAT and SAT depots in between normoglycemia and hyperglycemia groups in different lipid fractions. A: Triacylglycerol (TAG), (B) diacylglycerol (DAG), (C) monoacylglycerol (MAG), (D) phospholipids (PL), (E) free fatty acids (FFA) and (F) cholesteryl esters (CE). Data presented as mean ± SEM were analyzed using two-way ANOVA followed by Tukey’s post hoc test if there was an interaction effect, ∗*P* < 0.05, ∗∗*P* < 0.01, ∗∗∗∗*P* < 0.0001. N = 13–15 per group.

The concentrations of DHA in TAG were higher in the SAT compared to the VAT depot (*P* = 0.0030) and higher by ∼30% in the hyperglycemia compared to normoglycemia (*P* = 0.0009) (Fig. 4A). No main effect of depot (*P* = 0.0584) or glycemia (*P* = 0.8893) for DHA were observed in DAG fraction (Fig. 4B). No main effect of depot was observed for DHA in the MAG (*P* = 0.9866) fraction; however, DHA concentrations were ∼21% lower in hyperglycemia glycemia group compared to normoglycemia glycemia group (*P* = 0.0111) (Fig. 4C). DHA in the PL fraction was higher in VAT compared to SAT (*P* = 0.0332, Fig. 4D). An interaction effect (depot × glycemia) was present for DHA in FFA (*P* = 0.0383) and CE (*P* = 0.0471) fractions (Figure 4E, F).

In addition to FA concentrations from the total lipid pool and six lipid fractions, relative FA percentages were also assessed to determine proportional changes within lipid pools. Briefly, the total lipid and TG fraction relative % data suggest ARA, EPA and DHA are also higher in the SAT depot compared to VAT (Supplemental Tables S2–S15).

### Plasma and adipose tissue inflammatory markers

In plasma, markers of inflammation and immune activation including CRP (*P* = 0.4801), MCP-1 (*P* = 0.9436), CD80 (*P* = 0.0932) and IL-8 (*P* = 0.8661) showed no differences between normoglycemic and hyperglycemic groups. Adiponectin (*P* = 0.0065) was higher in the normoglycemic compared to hyperglycemic group (Supplemental Table S16). For the AT depots, no interaction was present for adiponectin (*P* = 0.6088), MCP-1 (*P* = 0.713) and IL-8 (*P* = 0.616). However, main effects of depot were present for adiponectin (*P* < 0.0001), MCP-1 (*P* = 0.0042) and IL-8 (*P* = 0.0412), indicating higher concentrations in the VAT depot compared to SAT. No main effects of glycemia were present for adiponectin (*P* = 0.493), MCP-1 (*P* = 0.9182) and IL-8 (*P* = 0.5459) (Supplemental Table S17).

## Discussion

To our knowledge, this is the first study to compare FA profile of total lipids and lipid classes between participant-matching SAT and VAT depot samples from individuals with obesity and varying glycemia levels, revealing distinct depot and glycemic-status patterns associated with metabolic health. Interestingly, the SAT depot contained higher levels of certain n-6 and n-3 PUFAs compared to the VAT, notably for ARA, EPA and DHA, which could be a reflection of reduced utilization or turnover changes leading to accumulation. Interestingly, plasma inflammatory markers were unchanged between normoglycemic and hyperglycemic groups. Only plasma adiponectin was higher in the normoglycemia group compared to hyperglycemia. AT exhibited depot-specific variation, with higher MCP-1 and IL-8 levels in VAT compared to SAT, supporting the notion that VAT demonstrates sustained levels of inflammation despite no differences in the plasma. Collectively, our work suggests that there are significant differences in PUFA content in SAT and VAT depots of individuals with obesity and hyperglycemia that may be related to changes in FA metabolism.

Despite not observing any differences in plasma FAs, our findings demonstrate tissue-specific lipid variation. Interestingly, we observed higher concentrations and relative % of certain n-6 and n-3 PUFAs in SAT depot compared to the VAT. This difference between plasma and AT FA profiles likely reflects the transient nature of circulating lipids, whereas AT depots more reliably capture longer-term (27) lipid storage metabolism, supporting its relevance as a matrix for assessing depot-specific lipid alteration in obesity. The higher levels of n-6 and n-3 PUFAs in SAT may suggest compositional enrichment along with greater lipid storage and altered remodeling capacity that is independent of circulating PUFA supply. Our findings are consistent with evidence from human females undergoing laparoscopic Roux-en-Y gastric bypass surgery, which demonstrated that 20-carbon PUFAs were consistently higher in the SAT compared to VAT depot, while essential FAs linoleic acid and α-linolenic acid did not differ between depots (28, 29). SAT functions as an important lipid storage depot and has reported to differ from VAT in metabolic activity, including differences in lipolytic activity and FA turnover (30, 31, 32, 33). In this study, higher concentrations of ARA, EPA, and DHA in SAT compared to VAT may reflect depot-specific differences in FA composition and regulation of lipid handling. Although differential FA retention, remodeling or turnover may contribute to these depot-specific patterns, these mechanisms remain speculative, as they were not directly assessed in the current study.

Within the total lipid pool, ARA and DHA concentrations were higher in the hyperglycemia group, suggesting that glycemic dysregulation may be associated with altered ARA and DHA metabolism, consistent with previous reports (34, 35). Although data on direct comparisons of ARA concentrations across varying glycemia levels remains limited, several studies have linked higher n-6 PUFAs, including ARA in AT to adverse metabolic health risk outcomes and possible metabolic AT dysfunction in individuals with severe obesity (36, 37). However, these data are not stratified across specific depots. Although ARA is a precursor to eicosanoids which are involved in pro-inflammatory pathways that may disrupt insulin signaling, higher SAT ARA levels may only reflect depot-specific differences in FA metabolism rather than direct inflammatory activity. Even though n-3 PUFA-derived mediators have pro-resolving properties, DHA was higher in the hyperglycemic group and EPA showed no glycemic differences. Both EPA and DHA can activate peroxisome proliferator activated receptors (PPARs) and regulate genes involved in lipid uptake, storage and metabolism (38, 39). While molecular mechanisms are widely known, significant gaps remain in understanding how these FAs may be altered in obesity and related metabolic complications, including insulin resistance. Individuals with obesity may have less incorporation of n-3 PUFAs in the membrane phospholipids, potentially limiting the availability of these FAs for the production of downstream lipid mediators such as eicosanoids and docosanoids (38) and increasing the likelihood of their accumulation as TAGs. These possible mechanisms may have contributed to the higher DHA retention in the hyperglycemic compared with normoglycemic group and in the SAT depot. However, there is a lack of alterations in EPA observed in our study warrant further investigation. Additionally, Schulte *et al.* reported higher levels of DHA-derived mediators in subjects with diabetes and morbid obesity compared to non-diabetic subjects with mild obesity. Therefore, highlighting differences in PUFAs based on glycemic status; however, these results were limited to serum, further highlighting the importance of investigating AT depots (40). Our findings add valuable insight to the current literature by demonstrating that ARA and DHA differences are evident within SAT depots and in varying glycemic status in obesity.

PUFA distribution across the six lipid fractions demonstrated that ARA concentrations were mostly higher in SAT compared to VAT depot across multiple lipid fractions including TAG, DAG, PL, FFA and CE. This indicates that depot differences in total ARA concentration are driven by changes across both storage and metabolically active lipid fractions (29). In terms of glycemic differences, ARA concentration was higher in the hyperglycemic compared to normoglycemic group in the DAG fraction. This is notable given the role of DAG in intracellular signaling; however, the extent to which FA composition influences its function as a second messenger in metabolic pathways remains unclear (41). Higher ARA in these fractions may reflect altered lipid remodeling or its retention in the presence of hyperglycemia despite being normolipidemic. In contrast, higher ARA in the normoglycemic group of MAG fraction may suggest differences in intermediary lipid turnover; however, as MAG also serves as a precursor in TAG synthesis, these findings could reflect variation in either lipolytic processing or re-esterification pathways between glycemic states (42). The observed higher ARA in the FFA fraction within SAT in our study is particularly interesting, as it may suggest that hyperglycemia influences ARA handling in a depot-specific manner. However, given the absence of an effect of glycemia, these findings likely reflect context-dependent changes, and the relationship between ARA and hyperglycemia appears potentially bidirectional, with precise mechanisms yet to be defined.

EPA showed differences in several lipid classes with higher concentrations in normoglycemia compared to hyperglycemia. It was higher in the normoglycemic group compared to hyperglycemic group in DAG, MAG and CE fractions, suggesting altered lipid partitioning. DAG and MAG are intermediates in TAG synthesis and lipolysis and their FA compositions reflects the ongoing lipid remodeling in the AT (43). To the best of our knowledge, there are no well-established human studies reporting positive association between higher presence of EPA in DAG fraction with glycemic status. Hypothetically, higher levels of EPA observed in normoglycemia compared to hyperglycemia in DAGs could be attributed to better insulin sensitivity. However, no direct work is available to help understand why higher levels of EPA were present in the normoglycemia group in DAG, MAG and CE fractions, highlighting the need for future studies. No glycemic effects were observed in the TAG fraction, which is the most abundant lipid fraction (44). This likely explains why overall EPA changes were not observed.

Higher DHA concentrations in the hyperglycemic group in TAGs, the storage form, may be reflective of its increased uptake-incorporation or decreased metabolism. It could also potentially be representing a compensatory mechanism to combat metabolic abnormalities in the hyperglycemic group. Additionally, DHA being higher in TAG may also be due to higher fat amount in the SAT depot. However, due to the nature of the study design, it is difficult to measure the total amount of SAT in the hyperglycemic group. Additionally, DHA levels in the total lipid pool were higher in the SAT depot; however, DHA in the PL fraction were higher in the VAT compared to the SAT depot. This may suggest potential production of DHA-derived lipid mediators originating from the VAT depot. Higher DHA in the MAG and CE fractions in the normoglycemic group could possibly be due to pro-resolving oxylipins. CEs have been reported to generate oxylipins via LOX-mediated action (45, 46, 47). The mechanisms of ARA, EPA and DHA in the specific lipid fractions based on glycemic status remain limited, especially in human AT, highlighting the need for exploring inflammatory lipid mediators.

### Strengths and limitations

This study has several strengths, including, to our knowledge, being the first to assess FA profile and six lipid classes in participant-matched human plasma, VAT and SAT depots in individuals with obesity and varying glycemic status. Without any dietary or supplemental interventions, this work enables direct insight into the FA composition of human AT depots. Furthermore, the analysis of six lipid classes allows for identification of specific FAs in each lipid pool, which offers a better understanding of depot-specific differences in lipid composition. However, these measurements remain static and do not capture dynamic metabolic processes such as FA turnover or flux. Future studies incorporating tracer-based approaches would offer a more comprehensive understanding of AT lipid metabolism.

A few limitations of this study should be considered when interpreting the findings. The study lacked AT samples from healthy controls and although the group categorization was based on fasting blood glucose, the hyperglycemia group exhibited relatively low HbA1c levels, indicative of mild glycemic dysregulation. This may have attenuated the ability to detect differences, despite the inclusion of HOMA-IR and adiponectin as additional metabolic measures. No sex specific assessments of FA composition were conducted due to lack of adequate male sample size, as well as relatively small overall cohort, which limited statistical power. Moreover, detailed information on lifestyle factors, genetic background, ethnicity, duration of obesity and hyperglycemia, along with dietary or medical interventions prior to bariatric surgery were not known, all of which may influence AT FA composition and inflammatory markers. In addition, perioperative factors, such as the duration of bariatric surgery and anesthesia, could influence AT lipid composition, particularly the FFA pool, and impact inflammatory markers. Although plasma FA composition did not differ between glycemic groups, correlations between plasma and AT FA composition were not assessed. Therefore, conclusions regarding the relationship between circulating and tissue-specific FA profiles should be interpreted cautiously. Lastly, as this is an observational study, the mechanisms underlying the observed differences in the FA composition cannot be determined.

In conclusion, we observed higher levels of ARA, EPA and DHA in the SAT depot compared to the VAT. Regarding glycemic status, ARA and DHA were higher in the hyperglycemic group, and the findings across the six lipid fractions were generally consistent with those observed in the total lipid pool. These findings suggest altered AT PUFA composition in hyperglycemia; however, the underlying metabolic mechanisms remain to be determined. Although no inflammatory differences were observed between normoglycemic and hyperglycemic individuals, differences in AT PUFA and FA composition remained evident. Altogether, these findings suggest that AT FA composition differs according to depot and glycemia status, even in the absence of detectable differences in plasma FA composition or inflammatory markers. Therefore, further investigations aimed at assessing PUFA-derived metabolites, along with direct assessments of FA turnover and metabolic flux are warranted to understand the mechanism underlying depot- and glycemia-based differences in AT FA composition in obesity.

## Data availability

All datasets generated and/or analyzed during this study are available from the corresponding author on reasonable request.

## Supplemental data

This article contains supplemental data.

## Conflict of interest

The authors declare the following financial interests/personal relationships which may be considered as potential competing interests:

A.T. received research funding from Johnson & Johnson, Medtronic, GI Windows and Bausch Health for studies on bariatric surgery. A. T. acted as consultant for Bausch Health, Novo Nordisk, Biotwin and Boehringer Ingelheim.
