# Supplementary material for: Adults with obesity and hyperglycemia have higher arachidonic and docosahexaenoic acid in subcutaneous adipose tissue
Source: J Lipid Res. 2026 Jul 17;67(8):101106. doi: 10.1016/j.jlr.2026.101106 (PMC13476625; doi:10.1016/j.jlr.2026.101106)
Supplement: Supplementary file 1 [file mmc1.docx]

**Supplemental Table S1.**

Plasma fatty acid concentrations and relative %. Data represented as nmol/ml ± SEM and % ± SEM. A student t-test was performed to determine significance between groups *p <0.05.

**Supplemental Table S2.**

Total FA concentrations.

**Supplemental Table S3.**

Total FA relative %.

**Supplemental Table S4.**

TAG concentrations.

**Supplemental Table S5.**

TAG relative %.

**Supplemental Table S6.**

DAG concentrations.

**Supplemental Table S7.**

DAG relative %.

**Supplemental Table S8.**

MAG concentrations.

**Supplemental Table S9.**

MAG relative %.

**Supplemental Table S10.**

PL concentrations.

**Supplemental Table S11.**

PL relative %.

**Supplemental Table S12.**

FFA concentrations.

**Supplemental Table S13.**

FFA relative %.

**Supplemental Table S14.**

CE concentrations.

**Supplemental Table S15.**

CE relative %.

**Supplemental Table S16.**

Plasma inflammatory markers including CRP, MCP-1, CD80, IL-8 and adiponectin. Data represented as pg/ml ± SEM. A student t-test was performed to determine significance between groups *p <0.05, **p < 0.01. N=14-15 per group.

**Supplemental Table S17.**

Adipose tissue inflammatory markers including adiponectin, MCP-1 and IL-8. Data represented as pg/mg of protein ± SEM and analyzed using two-way ANOVA followed by Tukey’s post hoc test if there was an interaction effect, *p <0.05, **p < 0.01, ****p < 0.0001. N=14-15 per group.
